# The Yellow Fever Epidemic

**Published:** 1878-10

**Authors:** 


### Editorial.

### The Yellow Fever Epidemic.

All Newspapers and most Medical Journals are filled with yellow fever reports or articles. The subject is so forced upon the profession and public that it is almost impossible to withhold expression on the subject. Few concise satisfactory facts concerning it have yet been stated; what it is, where it originates, what laws (if any) it observes, how it is propagated, and by what influences it is controlled, does not yet fully appear.

The editor of the *Scientific American* says: "It comes in every case from without; there is no spot in the United States where it is indigenous. Its birth-places are the West Indies, the South American coast, and, possibly Vera Cruz in Mexico. From these countries it invades, almost every summer, our sea-board cities, and occasionally produces a desolation such as words fail to describe. This disease made its first appearance in this country in 1768, and from that time down to 1877 it had visited us seven hundred and forty-one times, spread its ravages to two hundred and twenty-eight cities and towns, and extended to twenty-eight States of the Union, causing sixty-five thousand three hundred and eleven deaths counted—besides the innumerable deaths of which no record was made. Of all these numerous appearances of the disease among us, forty-five per cent. are directly traceable to foreign origin."

Efficient National Quarantine and thorough disinfection are spoken of as our only protection against cholera and yellow fever visitations, and we should all feel a degree of relief, if we could be sure, that in either or both of these we could look for safety.

The question arises, how is it that in some cities the disease prevails most extensively, and proves most fatal, in the best, highest, cleanest, and to all appearance the most hygienically perfect districts of the town or city? Our understanding of hygienic law is pretty severely tested, sometimes by visitations of epidemic disease—our boastings of knowledge in these mattesr proving vain. After all it must be hoped that disinfection and quarantine will be able to effect some good.

One word about our internal quarantine. It seems possible to so institute quarantine regulations as to protect the importation of the disease, but when it is imported and spreading terror and dread into all the families of a great city, who at once attempt to flee the town, going in every possible direction, occupying every route of exit, not because they have fever but because they fear they may have it, their friends and neighbors sick or dead, and possibly some of their own families now scarcely able to go with them, it would seem to be quite otherwise. In this condition of distress they attempt to escape the plague, as they have no better name for it. Now can quarantine prevent this stampede to adjoining towns. Are we to stop a gentleman's private carriage containing himself and family, and institute quarantine regulations, can physicians say that the craft is loaded with yellow fever, and must stop; and suppose it is examined and reported free from disease, is it certain that two weeks later, the usual period of incubation having expired, the whole ship's crew and passengers may not develope the plague, can quarantine do any thing in such cases to protect surrounding districts? If Memphis is destroyed, must not the surrounding country also suffer? Quarantine and disinfection, however well directed and inforced, do not make us feel quite safe, and there is probably a "great deal of danger in being safe" or sure in this matter of epidemic disease.

If towns and cities will not listen to repeated warning and neglect all sanitary law, (if sanitary science has really reached any law,) then visitations of "the plague" in some form is to be expected.

Continuous high temperature is believed to induce yellow fever in cities prepared for it by hygienic neglect. Dr. Comstock, of St. Louis, speaks of it as essential to the epidemic form of the disease.

At a distance from the fearful destruction of life and commercial loss already incurred, and after reading what is said about it by most careful observers, the conclusion is irresistable, that as Physicians, Hygienists, Sanitarians, &c., &c., we are yet very unsettled and ignorant as to all the essentials concerned in the origin and spread of yellow fever. It yet seems to be a subject open for and demanding investigation at this very opportune time when all are so much interested in every thing connected with yellow fever and epidemic disease generally.

We subjoin a quotation from Dr. Bell, editor of the *Sanitarian*, containing valuable and suggestive facts on this subject:

That yellow fever has not prevailed in New York and Brooklyn, and several other cities north of its more usual latitudes, the past summer, is not by any means due to certain acts of inhumanity which have been committed against the sick afflicted with it, and the well flying from it, but to the good fortune of these cities in that while they tolerate the local conditions, they have not been subject to a continuous high temperature for several weeks—above seventy-five degrees—also necessary to it. But the present exemptio is no guaranty for the future. So long as people tolerate the local conditions, they continue to risk the recurrence of an exceptional season of high temperature with results the same as have been in the past.

Yellow fever is eminently portable to places of like conditions as those in which it originates by means of *fomites*—clothing, books, dunnage, certain articles of merchandise, ballast; and, above all, by filthy vessels—packed up or closed in, in the places where and when the disease is prevailing; hence the necessity of quarantine of such materials until they are disinfected. But inasmuch as the disease is *not personally contagious*—is not communicable from one person to another—persons should in all cases be allowed their freedom, no matter what the degree of exposure to which they have been subject. And during the prevalence of an epidemic in any place, everybody should be encouraged and aided, as far as practicable, to escape from it as the surest means of safety.

The sick with yellow fever should be cared for under such circumstances as will most conduce to their recovery; and as they are never dangerous to the surroundings, *cannot* give the disease to any other person or thing, to remove such sick persons from healthy places, or take them from the care of their friends to out-of-the-way places, or, as has been practised in New York and Brooklyn, take them to the Quarantine Hospital, is alike calculated to render such cases more dangerous, and to alarm the public on a false issue.

In illustration of its non-contagiousness: during the naval service in the Mexican war the writer messed and slept in a room of about 8,000 cubic feet capacity, but sufficiently open for the wind to blow through it, with from five to thirteen yellow fever patients for his companions for several weeks; and in an adjoining room belonging to the same building (the Salmadino Hospital, wholly built of unplaned pine boards, on an island near Vera Cruz), with an open door-way between, containing at no time less than forty, and for a large portion of the time over a hundred other cases, *without taking the disease.* But subsequently, at the end of one week spent on board an infected ship, he was stricken with it.

Again: at Fort Hamilton, in 1856, while attending Dr. Joseph H. Baily, of the United States Army, with yellow fever, and fearing the worst results from a relapse which overtook him in that then badly infected place, the writer had him carefully transferred on a bed in an easy wagon to the family of a brother, and the nursing care of his devoted wife, in Fourteenth street, New York, for which the public was none the wiser nor the worse. Dr. Baily and those who took care of him still live to rejoice over the result. It is almost needless to add nobody was made sick by the "risk."

In the New York Quarantine, while in charge of the yellow fever hospital ship—in the lower Bay, in 1862 and with yellow fever patients on board—the writer took his wife and young children on board for aeration, which they much enjoyed. And there he also permitted the devoted wife of a naval officer to watch over and bathe the brow of her husband during several days while he was *in extremis* with black vomit, to hold his hand at death, and to attend the burial. Thence she returned for a few days to the Mansion House, Brooklyn; and to her home. Nothing but good ever came of it.

Of good riddance, and the effect of freedom from an infected place: During the same service in Quarantine, about the middle of September, the United States steamer "Delaware," belonging to the Quartermaster's Department, arrived from Port Royal with over one hundred of crew and invalid soldiers on board, infected with yellow fever. Three died on the voyage; three cases were sent to the Floating Hospital; and the great danger of forty-six invalid soldiers, and more than as many more of other persons who had not had the disease, requiring immediate relief, was reported to the Commissioners of Quarantine. Three days having been fruitlessly spent in an effort to charter a hulk to which those well persons might be transferred, to cover the bugaboo period of "incubation," and, meanwhile, from three to seven new cases occurring daily, the writers took the responsibility of having every person on board, except necessary ship-keepers, washed, and dressed in clean suits, and sent to the Floating Hospital. There he kept them, though in rather close quarters (with due allowance for the sick) for a week, by the end of which time a transfer vessel was obtained, on board which they were placed and kept (not by the advice or authority of the writer) for two weeks longer; but *from the time they were removed from the infected ship "Delaware" not another case occurred among them.* So much for elimination as against development.

